# Association between Lung Fluid Levels Estimated by Remote Dielectric Sensing Values and Invasive Hemodynamic Measurements

**DOI:** 10.3390/jcm11051208

**Published:** 2022-02-23

**Authors:** Teruhiko Imamura, Masakazu Hori, Yohei Ueno, Nikhil Narang, Hiroshi Onoda, Shuhei Tanaka, Makiko Nakamura, Naoya Kataoka, Mitsuo Sobajima, Nobuyuki Fukuda, Hiroshi Ueno, Koichiro Kinugawa

**Affiliations:** 1 Second Department of Internal Medicine, University of Toyama, 2630 Sugitani, Toyama 930-0194, Japan; masahori6059@yahoo.co.jp (M.H.); fef6ge@gmail.com (Y.U.); ohiro0203@gmail.com (H.O.); stanaka@med.u-toyama.ac.jp (S.T.); nakamuramk1979@gmail.com (M.N.); nkataoka@icloud.com (N.K.); soba1126@yahoo.co.jp (M.S.); nfukuda@med.u-toyama.ac.jp (N.F.); hueno@med.u-toyama.ac.jp (H.U.); kinugawa-tky@umin.ac.jp (K.K.); 2 Advocate Christ Medical Center, Oak Lawn, IL 60453, USA; nikhil.narang@gmail.com

**Keywords:** congestion, heart failure, hemodynamics, ReDS

## Abstract

Background: Remote dielectric sensing (ReDS™) is an electromagnetic-based technology used to noninvasively measure lung fluid levels. The association between ReDS values and invasively measured hemodynamics, particularly among those with small physics, remains unknown. Methods: Consecutive patients with chronic heart failure who were admitted to our institute and underwent invasive right heart catheterization as well as simultaneous ReDS measurement at clinically stable conditions between September and November 2021 were prospectively included. The colinearity between ReDS values and pulmonary capillary wedge pressure was studied. Results: In total, 30 patients (median 79 (73, 84) years old, 13 men) were included. Median ReDS value was 26% (22%, 28%). ReDS values had a moderate collinearity with pulmonary capillary wedge pressure (r = 0.698, *p* < 0.001), even among those with a body height < 155 cm. ReDS values with a cutoff of 28% predicted a pulmonary capillary wedge pressure > 15 mmHg with sensitivity 0.70 and specificity 0.75. Conclusions: An electromagnetic-based engineering ReDS might be a potential tool to estimate cardiac pressure in patients with heart failure, including those with small physics.

## 1. Introduction

Patients with chronic heart failure are at risk for downstream morbidity without optimization of medical therapy. Furthermore, the use of novel engineering may offer further clinical benefit in those with persistent congestion, where quantification of intra-cardiac filling pressures can be estimated and used as a surrogate to tailor medical therapies [1]

Recently, remote dielectric sensing (ReDS™, Sensible Medical Innovations Ltd., Netanya, Israel) system, which is a novel electromagnetic-based engineering to quantify lung fluid volume noninvasively, has been clinically introduced [2]. One prior analyses observed a significant collinearity between ReDS value and lung fluid level measured by high resolution computed tomography [3], while other contemporary analyses demonstrated the advantage of ReDS in guiding heart failure management [4,5]

However, the association between lung fluid volume quantified by ReDS and intra-cardiac pressures measured by invasive right heart catheterization, particularly among those with smaller physics, remains unclear [6]. Clinical utility of ReDS system to those with smaller body size should also be demonstrated particularly for the Asian population, who had relatively smaller physics. In this study, we studied the collinearity between ReDS value and invasively measured hemodynamics among a Japanese chronic heart failure cohort.

## 2. Methods

### 2.1. Participant Selection

Consecutive patients who were admitted to our institute for worsening heart failure and received right heart catheterization to assess hemodynamics after hemodynamic stabilization between September and November 2021 were included in this single-center prospective study. Patients with mechanical circulatory support, unstable clinical condition including cardiogenic shock, or too large of body habitus to wear the ReDS system were excluded. The local ethical review board assented the present study and all of the participants signed informed consent beforehand.

### 2.2. Study Protocol

Invasively measured hemodynamics, ReDS values, and plasma B-type natriuretic peptide levels were all measured on the same day for all of the participants. Hemodynamic data including pulmonary capillary wedge pressure (PCWP) were measured using right heart catheterization in a standard manner by the heart failure experts. All of the procedures were performed via external jugular vein. All data were obtained at an end-expiratory timing. The procedures were performed and the obtained data were read by clinicians who were blinded on patients’ ReDS values. ReDS values were measured just before right heart catheterization, as detailed below.

### 2.3. ReDS System

**Figure 1:**
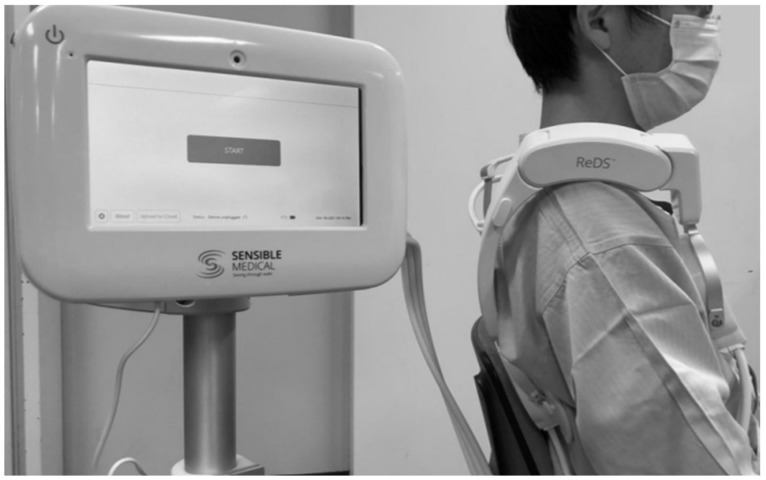
A ReDS system consisting of a monitor and a sensor unit.

ReDS engineering was previously detailed [2]. In brief, ReDS estimates the percentage of lung fluid volume, estimating the degree of pulmonary congestion. ReDS employs low-power electromagnetic signals emitted between two sensors embedded in wearable devices (Figure 1). The analyzed signal reflects the dielectric properties of the lung tissues between the sensors. The dielectric coefficient of a material is expressed by a frequency-dependent complex number describing its interaction with electromagnetic energy.

### 2.4. Statistical Procedures

We performed Shapiro-Wilks test for all of the continuous variables and confirmed their normal distribution. Variables with non-normal distribution were converted to common logarithm. Nevertheless, given small sample size, we exhibited all of the continuous variables as median and interquartile. Categorical variables were exhibited as numbers and percentages. The primary aim of the study was the quantify association between ReDS value and PCWP. Secondary aims included the association between ReDS value and other clinical parameters such as mean right atrial pressure and plasma B-type natriuretic peptide level.

Correlations were assessed using Pearson’s correlation coefficient. The impact of ReDS value on the PCWP level was investigated by linear regression analyses adjusting for 4 potential confounders irrespective of their statistical significance (age, body mass index, chronic kidney disease, and left ventricular ejection fraction). Receiver operating characteristics analyses with a Youden test were performed to determine the ReDS value which best estimated a PCWP > 15 mmHg.

All of the calculations were performed in SPSS Statistics 23.0 software (IBM Corp, Armonk, NY, USA) and two-sided *p* values less than 0.05 were considered significant.

## 3. Results

### 3.1. Baseline Characteristics

In total, 30 patients were included. Detailed baseline characteristics are displayed in Table 1. Median age was 79 (73, 84) years old and 13 (43%) were men. Almost half of the patients (57%) had body height ≤ 155 cm. Median left ventricular ejection fraction was 54% (42%, 66%) and median common logarithm of plasma B-type natriuretic peptide level was 2.22 (1.90, 2.47) pg/mL.

### 3.2. Association between ReDS Values and PCWP

**Figure 2:**
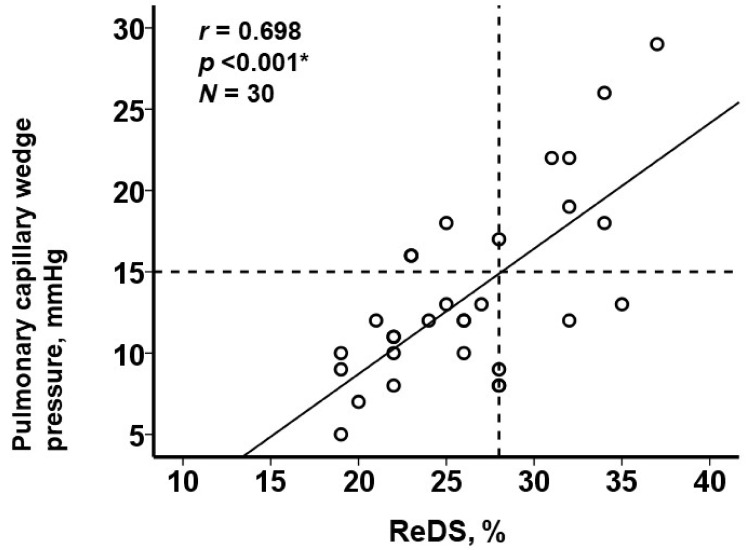
Relationship between ReDS values and PCWP values. A vertical line (ReDS value 28%) indicates a cutoff to predict PCWP > 15 mmHg. * *p* < 0.05 by Pearson’s correlation coefficient.

Median ReDS value was 26% (22%, 28%) and median PCWP was 12 (10, 17) mmHg. There was a moderate correlation between the ReDS value and PCWP (r = 0.698, *p* < 0.001; Figure 2). The ReDS value was independently associated with the levels of PCWP adjusted for 4 potential confounders (*p* = 0.001; Table 2). R2 value in this model was 0.54.

**Figure 3:**
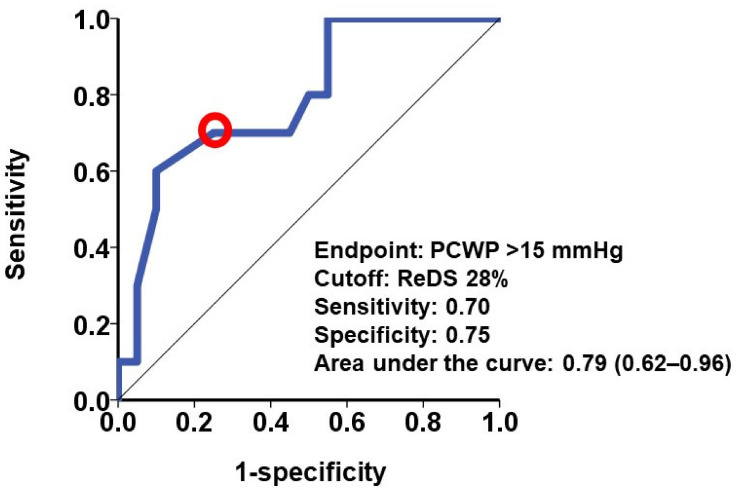
Receiver operating characteristics analysis for ReDS values to estimate PCWP > 15 mmHg. A red circle indicates the cutoff of ReDS value.

**Figure 4:**
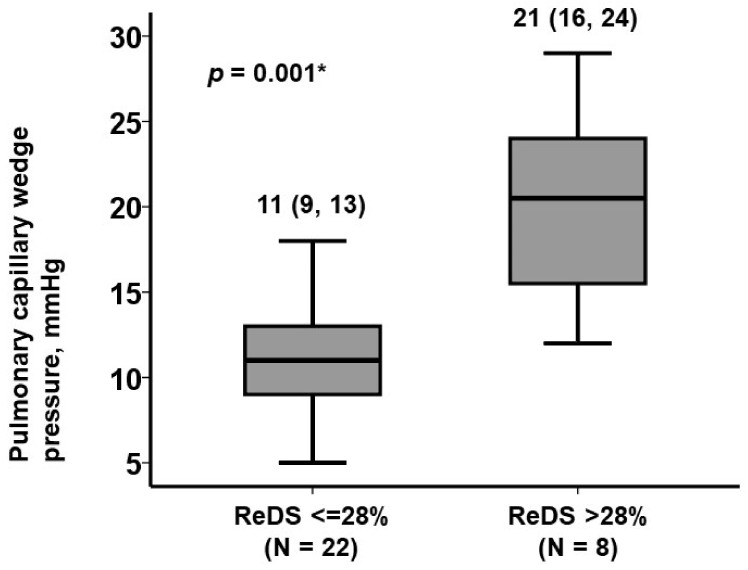
PCWP levels stratified by the cutoff of ReDS 28%. * *p* < 0.05 by Mann-Whitney U test.

A cutoff of a ReDS value to estimate PCWP > 15 mmHg, indicative of an abnormally elevated filling pressure, was 28% with a calculated sensitivity 0.70 and specificity 0.75 (Figure 3). Patients with ReDS values >28% (N = 8) had significantly higher PCWP levels than those with ReDS values ≤28% (N = 22) (Figure 4).

### 3.3. Association between ReDS Values and Other Parameters

**Figure 5:**
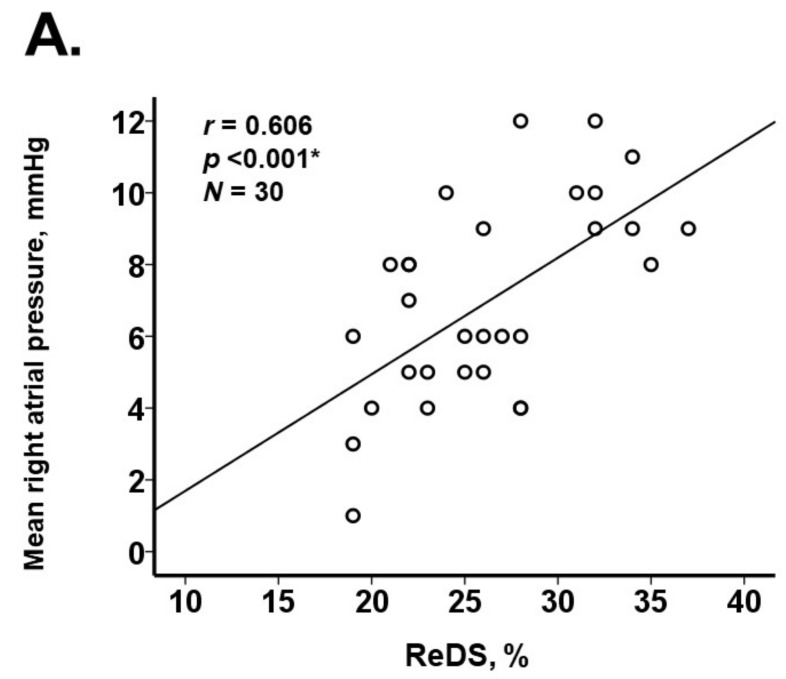

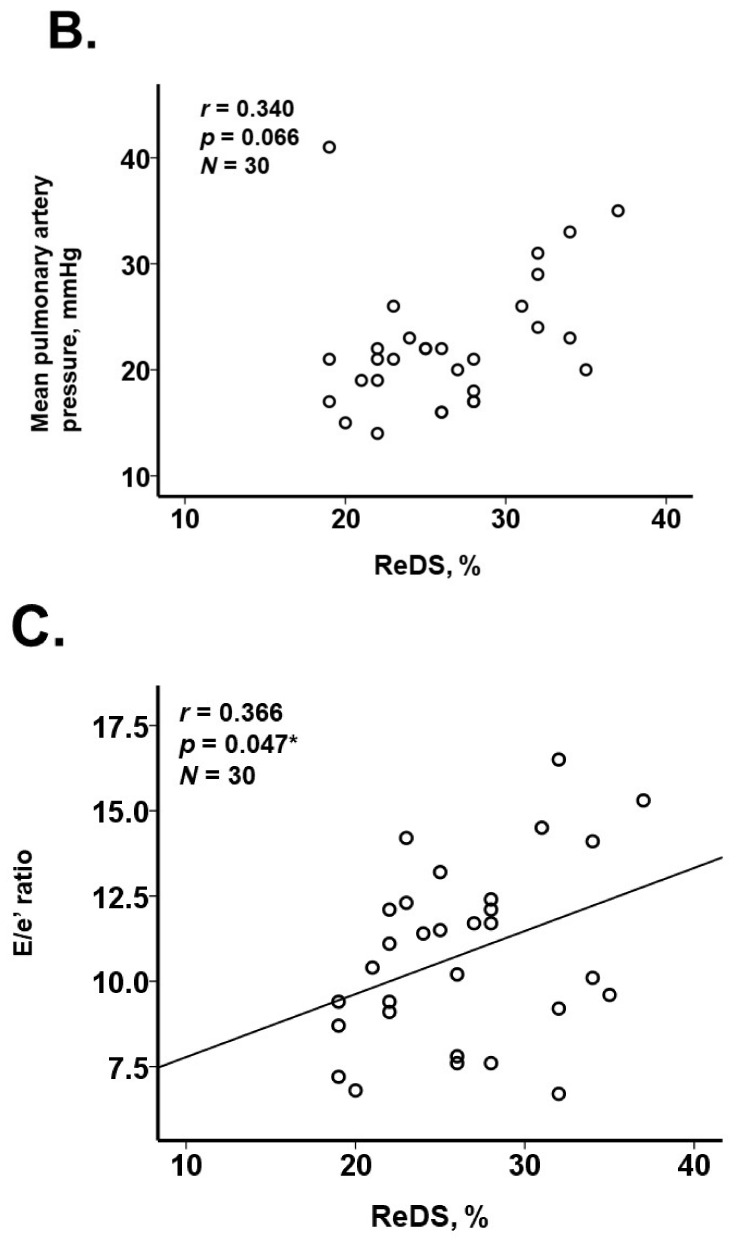

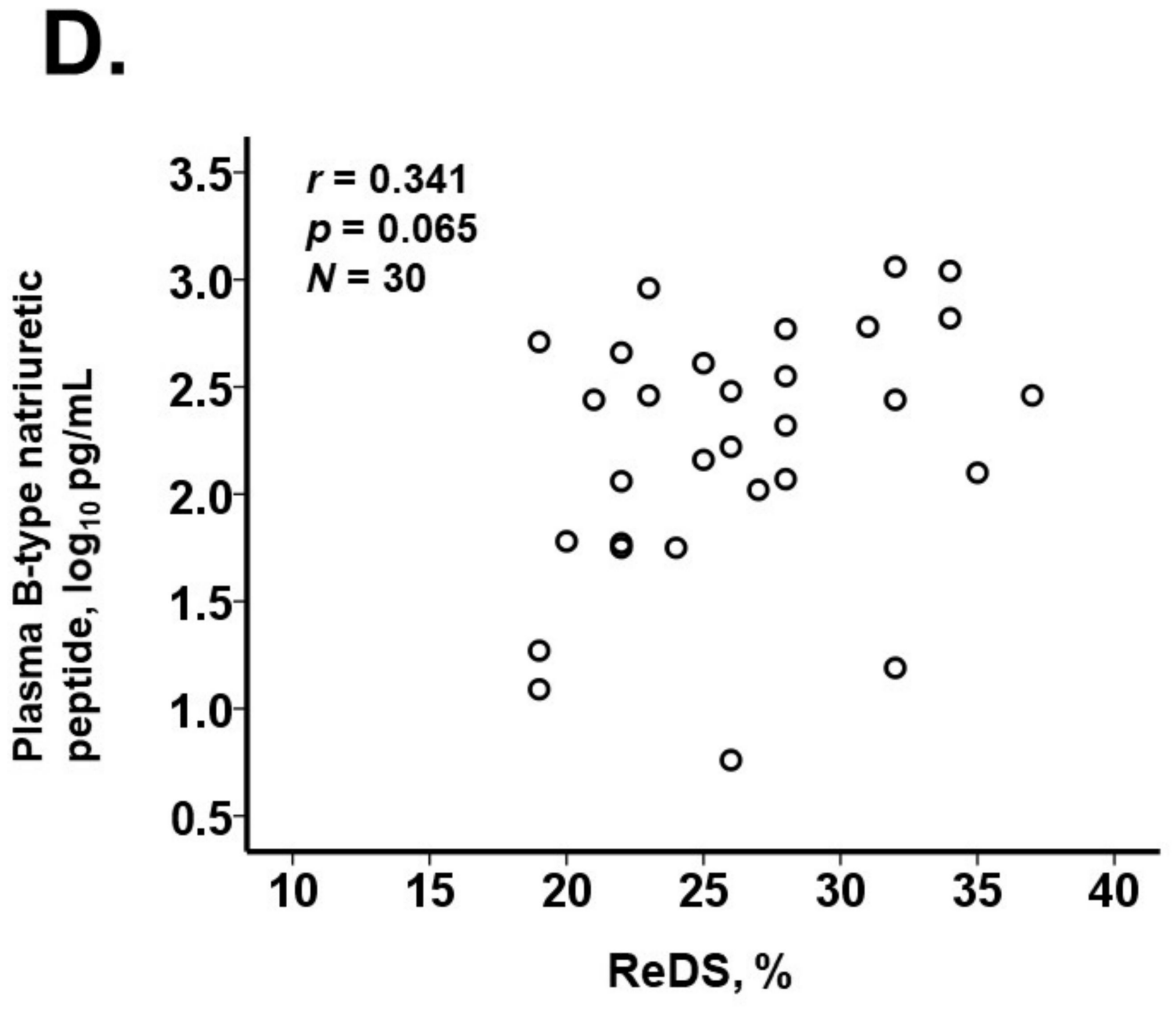
Correlation between ReDS values and other clinical variables including mean right atrial pressure (**A**), mean pulmonary artery pressure (**B**), E/e’ ratio (**C**), and plasma B-type natriuretic peptide (**D**). * *p* < 0.05 by Pearson’s correlation coefficient.

There was a moderate collinearity between ReDS values and mean right atrial pressure (r = 0.606, *p* < 0.001; Figure 5A). There was a weak correlation between ReDS value and mean pulmonary artery pressure (r = 0.340, *p* = 0.066; Figure 5B) and between ReDS value and E/e’ ratio (r = 0.366, *p* = 0.047; Figure 5C). The ReDS weakly correlated with plasma B-type natriuretic peptide levels (r = 0.341, *p* = 0.065; Figure 5D). Plasma B-type natriuretic peptide levels had a significant correlation with PCWP (r = 0.467, *p* = 0.009).

### 3.4. Sub-Group Analyses According to the Body Height

**Figure 6:**
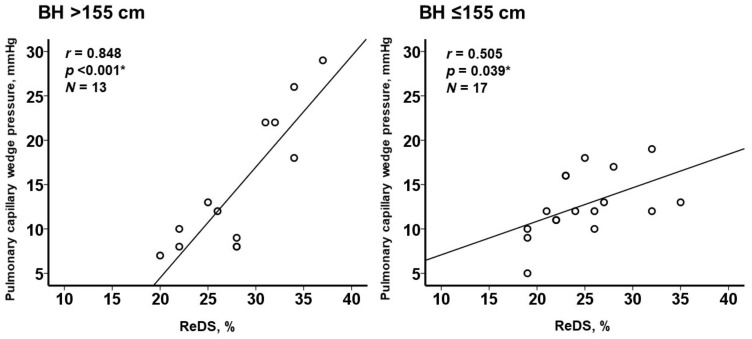
Correlation between ReDS value and PCWP stratified by body height. * *p* < 0.05 by Pearson’s correlation coefficient.

The collinearity between ReDS values and PCWP was significant among those with body height > 155 cm (N = 13) and those with body height ≤ 155 cm (N = 17), respectively, (*p* < 0.05 for both; Figure 6).

## 4. Discussion

In this proof-of-concept prospective study, we assessed the association between ReDS values, representative of lung fluid volume, and PCWP. The main findings were as follows: (1) there was a moderate collinearity between ReDS values and PCWP values irrespective of several potential confounders; (2) a positive correlation remained among those with small physics; (3) ReDS values moderately correlated with mean right atrial pressure and had a weak but non-significant correlation with plasma B-type natriuretic peptide levels.

### 4.1. ReDS Engineering

An accurate assessment of pulmonary congestion is challenging given the lack of proven techniques to quantify lung fluid levels in granular detail [7] Chest X-ray, echocardiography, and computed tomography, as well as physical examination, are generally used to assess the pulmonary congestion, though they are imprecise and prone to wide variation in clinical interpretation [8].

ReDS is a recently introduced engineering that quantifies lung fluid amount [2]. In patients with and without heart failure, ReDS system was non-inferior in estimating lung fluid amount compared to high resolution computed tomography [3]. ReDS-guided diuretic dose adjustment was a feasible strategy in patients with chronic heart failure [4,5]. Although large-scale analyses comprised of heterogeneous clinical cohorts are lacking, the ReDS engineering may be a hopeful procedure for non-invasive assessment of lung fluid amount.

### 4.2. ReDS Values and PCWP Values

There was a moderate collinearity between ReDS values and PCWP. Although we should not equate that the presence of lung volume as a direct representation of intra-cardiac pressure, the ReDS system might be a hopeful non-invasive alternative to estimate intra-cardiac pressures without performing right heart catheterization.

### 4.3. Prior Analyses

Similar findings were reported by Uriel and colleagues [6]. They proposed a cutoff of ReDS value 34% to estimate PCWP ≥ 18 mmHg. Of note, all of the participants in their study had body height >155 cm, which represents a common physics of the Western cohort, and the applicability of their findings to those with smaller physics remained uncertain.

In our study consisting of smaller physics, a cutoff ReDS of 28% was associated with PCWP > 15 mmHg, which represents another cut point of clinically significant congestion in patients with chronic heart failure [9]. Of note, the newly proposed cutoff of ReDS 28% was lower than the upper limit of the manufacturer-recommended cutoff of 35% (manufacturer-recommended normal rage was between 20% and 35%).

### 4.4. Body Size

Our cohort differed from prior studies as it relates to body size: median body mass index in our study was 22.9 whereas the mean body mass index in the study by Uriel et al. was 28.1 [6]. In their study, all of the participants had body height >155 cm and body mass index >22.0. Given all previous studies regarding the ReDS system were published in Western countries, the applicability of their findings to those with smaller physics is unknown. In this study, a significant correlation was observed even among those with smaller body size.

### 4.5. ReDS System and Right Heart Catheterization

We should reference both the ReDS system and right heart catheterization for a comprehensive assessment of clinical congestion in patients with heart failure. Of note, there are several patients with discordance between the two values, which may be explained by the differences in pulmonary vasculature and lymphatics in patients with chronic heart failure that in turn may affect the balance of intravascular and extravascular fluid volume. For example, some patients with low cardiac output with hypovolemia would have high PCWP and B-type natriuretic peptide levels but low ReDS. Cardiac unloading would be a suggested treatment, whereas aggressive diuretics therapy would rather decrease preload and cause cardiogenic shock.

### 4.6. Study Limitations

This study was comprised of a small sample size. Of note, our cohort was non-Western patients with small body size. Systolic function was relatively preserved in most patients. Applicability of our findings to those with other races and/or advanced systolic heart failure remains unclear. The ReDS system cannot distinguish other lung-occupying lesions including pneumonia and malignancies. We performed right heart catheterization following medical stabilization. Patients with decompensated heart failure in the acute phase during a period of aggressive diuresis were not studied. The existence of pulmonary congestion is more clinically obvious rendering the use of this technique less necessary, whereas situations of unclear clinical assessment could benefit from the ReDS system. We included variables that were considered to be potential confounders in the multivariable model. Some uninvestigated confounders might have been missed.

## 5. Conclusions

A non-invasive electromagnetic-based technology ReDS may be a hopeful procedure in estimating intra-cardiac pressures in patients with chronic heart failure, even among those with small body size.

## Figures and Tables

**Table 1:** Baseline characteristics.

	N = 30
Demographics	
Age, years	79 (73, 84)
Men	13 (43%)
Body height, cm	153 (149, 163)
Body height < 155 cm	17 (57%)
Body weight, kg	55.0 (50.1, 61.1)
Body mass index	22.9 (20.0, 24.5)
Comorbidity	
Hypertension	22 (73%)
Dyslipidemia	13 (43%)
Diabetes mellitus	8 (27%)
Atrial fibrillation	12 (40%)
Chronic kidney disease	18 (60%)
History of stroke	2 (7%)
History of coronary intervention	2 (7%)
Valvular disease	14 (47%)
Echocardiography	
Left ventricular end-diastolic diameter, mm	48 (44, 54)
Left ventricular ejection fraction, %	54 (42, 66)
Left atrial diameter, mm	45 (40, 51)
E/e’ ratio	10.8 (9.1, 12.3)
Hemodynamics	
Heart rate, bpm	70 (64, 82)
Mean right atrial pressure, mmHg	7 (6, 9)
Mean pulmonary artery pressure, mmHg	21 (19, 23)
Pulmonary capillary wedge pressure, mmHg	12 (10, 17)
Cardiac index, L/min/m^2^	2.1 (1.9, 2.3)
Medications	
Beta-blocker	16 (53%)
Angiotensin converting enzyme inhibitor	22 (73%)
Mineralocorticoid receptor antagonist	11 (37%)
Loop diuretics	15 (50%)
Plasma B-type natriuretic peptide, log_10_ pg/mL	2.22 (1.90, 2.47)
Remote dielectric sensing, %	26 (22, 28)

Continuous variables were stated as median and interquartile. Categorical variables were stated as number and percentage.

**Table 2:** Impact of Remote dielectric sensing values and other potential confounders on pulmonary capillary wedge pressure.

	Univariate Analysis		Multivariate Analysis	
	Beta Value (95% CI)	*p* Value	Beta Value (95% CI)	*p* Value
Age, years	0.06 (−0.17–0.30)	0.58	−0.004 (−0.22–0.21)	0.98
Body mass index	−0.21 (−0.78–0.35)	0.44	−0.22 (−0.79–0.13)	0.15
Chronic kidney disease	−3.58 (−0.78–0.63)	0.092	0.04 (−4.10–5.10)	0.82
Left ventricular ejection fraction, %	−0.08 (−0.20–0.04)	0.17	−0.02 (−0.11–0.11)	0.99
Remote dielectric sensing, %	0.77 (0.47–1.08)	<0.001 *	0.74 (0.38–1.26)	0.001 *

CI, confidence interval. * *p* < 0.05 by linear regression analysis.

## Data Availability

Data are available upon reasonable request.
